# New Algorithms for Computing the Time-to-Collision in Freeway Traffic Simulation Models

**DOI:** 10.1155/2014/761047

**Published:** 2014-12-31

**Authors:** Jia Hou, George F. List, Xiucheng Guo

**Affiliations:** ^1^School of Transportation, Southeast University, No. 2 Sipailou, Nanjing 210096, China; ^2^Department of Civil, Construction, and Environmental Engineering, NCSU, Raleigh, NC 27695, USA

## Abstract

Ways to estimate the time-to-collision are explored. In the context of traffic simulation models, classical lane-based notions of vehicle location are relaxed and new, fast, and efficient algorithms are examined. With trajectory conflicts being the main focus, computational procedures are explored which use a two-dimensional coordinate system to track the vehicle trajectories and assess conflicts. Vector-based kinematic variables are used to support the calculations. Algorithms based on boxes, circles, and ellipses are considered. Their performance is evaluated in the context of computational complexity and solution time. Results from these analyses suggest promise for effective and efficient analyses. A combined computation process is found to be very effective.

## 1. Introduction

The time-to-collision (TTC) has often been used as a risk assessment metric for traffic safety analyses. In freeway simulation models, TTC is often a critical element of a driver's trajectory management decision-making process. TTC assesses the interaction intensity among vehicles. However, computing the TTC is not trivial. Projections of future interactions among vehicles involve creating predicted trajectories for the subject vehicle as well as all other vehicles with which interactions might occur in order to see if collisions might occur. The objective of this paper is to consider and evaluate algorithms that could be used to compute this new TTC in microsimulation based models. The algorithm must be as efficient since the speed of the TTC computation will influence the capacity of the simulation model. In contrast with existing research, the algorithms discussed here address the problem from a 2D continuous perspective. Given that focus, several approaches are examined for computing the TTC in a fast and accurate manner. The results of these investigations are presented and one procedure is recommended for use.

Following this introduction, the next section reviews related research. The third part illustrates the problem of interest and the fourth presents several computational algorithms that are described. After that, the algorithms are analyzed and compared. This leads to a result that one of the algorithms is recommended as the primary methodology to apply in traffic simulation models.

## 2. Literature Review

The idea of computing a time-to-collision (TTC) was first suggested by Hayward [1]. He defined it as “the time required for two vehicles to collide if they continue at their present speed and on the same path.” Hydén suggested that lower TTC values correspond to higher conflict severities [2]. Although this point has been argued in the safety assessment literature, it seems clear that lower TTC values correspond to a higher probability of collision [3, 4]. Hence, TTC is generally perceived to be a primary and efficient measure in traffic safety assessment especially in assessing conflicts. In microscopic simulation, TTC is one of the most common safety surrogate assessment measures employed. In 2003, FHWA released a report that introduced the use of traffic simulation models to obtain surrogate safety measures [5]. In that document, computational algorithms for calculating surrogate safety measures for different conflict types were described, and example diagrams were provided to illustrate the calculations graphically. In 2008, FHWA combined traffic simulation and automated traffic conflict analysis to develop a software utility referred to as a surrogate safety assessment model (SSAM) [6].

Besides being a safety indicator, the use of TTC as a cue for decision-making in traffic has been suggested by several studies. For example, Horst found that both the decision to start braking and the control of the braking process itself can be based on TTC-related information [7]. By measuring drivers' last second braking and steering while approaching a surrogate target lead vehicle, a model is built which indicates that the drivers' response is according to an inverse time-to-collision threshold that decreases linearly with driver speed [8].

TTC will not be simply replaced by headway since field data showed that headway and TTC are independent of each other [9]. In the case of the TTC evaluations commonly performed today, vehicles are assumed to stay in the same lane until their positions overlap. As a result, the TTC calculations are one-dimensional and the kinematic variables are scalars instead of vectors. The time-to-collision of a vehicle-driver combination *i* at instant *t* with respect to a leading vehicle *i* − 1 on the same path is calculated with

(1) $$\begin{array}{c} \text{TT}{\text{C}}_{i}=\frac{X_{i}\left(t\right)-X_{i-1}\left(t\right)-l_{i}}{{\dot{X}}_{i}\left(t\right)-{\dot{X}}_{i-1}\left(t\right)}\;\forall {\dot{X}}_{i}\left(t\right)>{\dot{X}}_{i-1}\left(t\right), \end{array}$$

where ${\dot{X}}_{i}$ denotes the speed, *X* the position, and *l* the vehicle length [10]. From (1) it can be seen that the TTC is usually computed for a specific path. Laureshyn et al. indicated that TTC could make continuous description during the road users' encounter process and provided a procedure for calculating TTC for one vehicle's side and another's corner point [11].

Unfortunately, these computational procedures do not align well with the way vehicles move. They do not jump from one lane to another. Moreover, additional vehicles are considered besides the one in front. If the TTC is to be used as a decision-making aid, it should address situations where vehicles are in the process of changing lanes. Moreover, acceleration should be included not just location and speed. This paper presents a new TTC definition as well as algorithms based on that definition with these thoughts in mind.

## 3. TTC as a Trajectory Management Aid

### 3.1. The Context of the Simulation System

In the new algorithms presented here, the vehicles are treated as being in a two-dimension plane. Each one is represented by a rectangle located at a specific spot in the *X*-*Y* plane. Each has a velocity and an acceleration, both of which are vectors.

There are no “lead vehicles” or “following vehicles.” Each “subject” vehicle interacts with its nearby vehicles. The subject vehicle's actions are consistent with three rules: (i) follow the vehicles in front, (ii) avoid collisions, and (iii) scale the intensity of the actions taken based on the numerical value of the TTC. That is, employ more intense actions for larger TTC values and vice versa.

**Figure 1:**
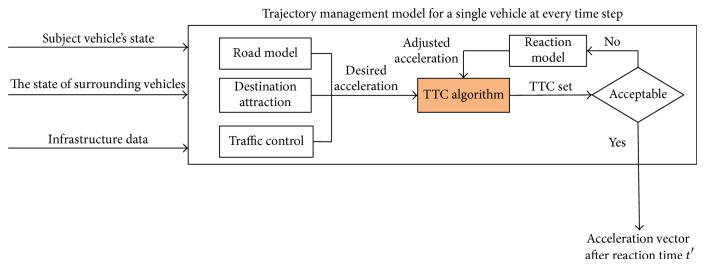
The TTC algorithm in the context of trajectory management.

The TTC is computed every time step for each vehicle pair that are close enough to each other that a TTC value is meaningful. The new coordinates of the “subject” vehicle are computed based on its old location, new speed vector, and new acceleration vector. Its new speed vector is similarly computed from its old speed and new acceleration vector. Acceleration vectors are determined through an analysis of the desired trajectory, road geometry, traffic controls (e.g., stop signs, traffic signals, and speed limits), and proximity to neighboring vehicles. Acceleration is deemed acceptable if it will not lead to any collisions. This process is illustrated in Figure 1. It places those computations into the broader trajectory management context. The “TTC algorithm” box highlights the materials being discussed in this paper.

### 3.2. A New Definition of TTC

The intent here is to compute a TTC that can influence the reaction maneuvers of subject vehicles. That is, the focus is not just on computing an “expected or actual time-to-collision.” Rather, it is to measure the imminent danger faced by the driver (the possibility that an accident will happen) if he/she continues on the current trajectory. The idea presented here is to define the TTC as “the time it will take a subject vehicle to collide with another vehicle in its immediate vicinity if the present trajectories continue to be followed.” This definition is different in two respects from the one currently in use: (i) acceleration is taken into consideration and (ii) the environmental objects include vehicles in other lanes as well as those in the same lane or the same path with subject vehicle. These differences allow the TTC to account for lane-changing maneuvers and to allow vehicles to follow trajectories that are not necessarily constrained by lane-based concepts (e.g., especially important for motorbikes, bicycles, and weaving vehicles).

### 3.3. Vehicle Locations and Buffer Areas

In the ideas presented here, all vehicles have an *X*-*Y* location in a 2D plane. This location is their centroid. Longitudinal motion is along the facility. Lateral motion is perpendicular. The vehicle's speed and acceleration are two-dimensional vectors and they operate from the centroid. The orientation of the rectangle and the speed and acceleration vectors need not be aligned.

Vehicles are represented by rectangles with specific lengths and widths. This contrasts with many of the existing traffic simulation models where vehicle dimensions are largely overlooked. In some cases, this is acceptable; in others it is not. In some circumstances (e.g., intersections), the relative speed is large enough that vehicles can be represented as particles. But on facilities like freeways, if the geometric details are ignored, errors can arise. In lane-changing maneuvers, for example, the necessary space for a vehicle of certain size is much larger than a simplified moving spot.

Technically, collisions occur if these rectangles overlap. Thus the TTC computational problem could be defined as a two-step process. First, determine interactions with nearby vehicles: given the subject vehicle's location, speed, and acceleration, how long will it be until its rectangular area overlaps that of another nearby vehicle. Second, find the minimum of these times. That value is the TTC.

**Figure 2:**
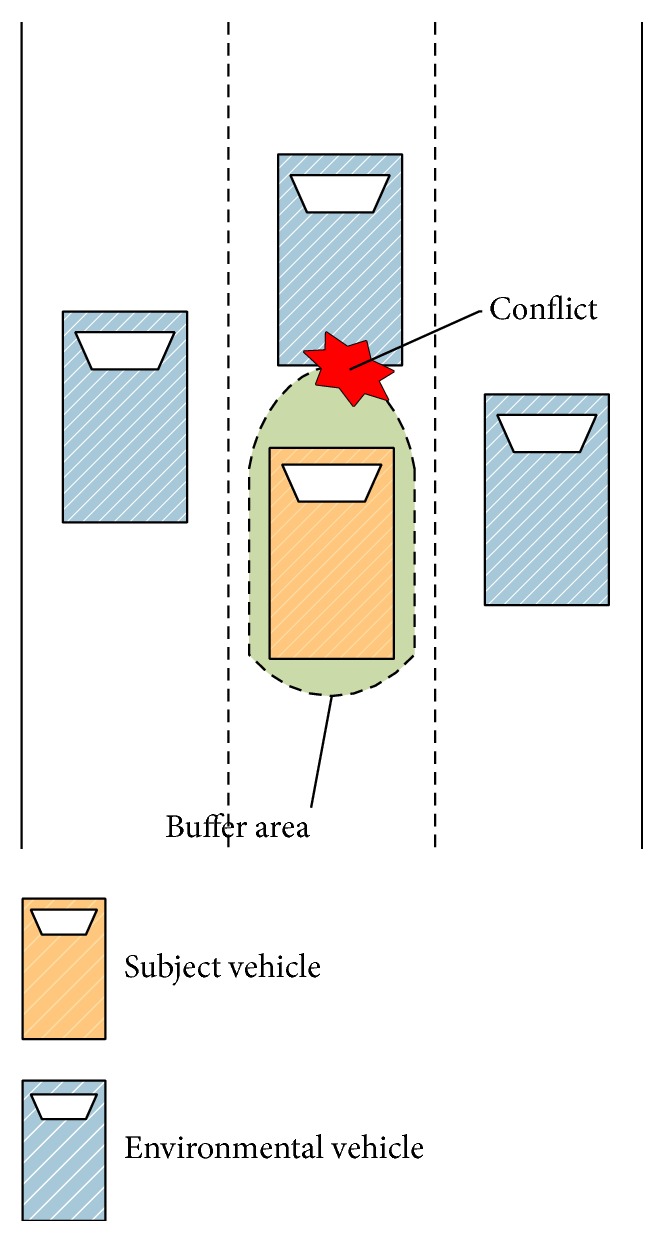
The demonstration of the buffer area.

In the context of this analysis, it is useful to introduce the idea of a buffer area around each vehicle, that is, an area that the vehicle occupies that encompasses not only its rectangular footprint, but a larger safety area. This idea is illustrated in Figure 2. The subject vehicle's buffer area is the green part around it.

The motivation for the buffer derives from the fact that drivers like to keep a certain safety distance from other vehicles. Drivers perceive invasion of this area by other vehicles as a conflict. In other words, the space range which the driver tries to protect from other vehicles is not only the vehicle itself but also the buffer area.

Moreover, the buffer does not have the same dimensions in every direction. The distance forward is larger than the distances left or right or rear. The safety distance of the center spot is larger than that of those spots on the right or left part because it takes more time to avoid conflict with an obstacle on the center point than that on the right or left part. Most maneuvers motivated by safety are going to result in keeping a safety distance rather than direct physical contact.

The extension of this buffer idea is to compute a TTC which is consistent with these driver perceptions. That is, make the “time-to-collision” consistent with the “time-to-conflict between the buffer areas.”

The size of the buffer should be based on driver behavior as observed in natural studies or driving simulators. As our algorithm is for the behavior model, the geometric measure of the safety buffer should be covered in the algorithm.

### 3.4. The Input and Output Issues

Before calculating the TTC, vehicle pairs which are close together need to be picked from the entire vehicle population. Although proximity does not change dramatically from one time step to the next, this assessment needs to be repeated each time step. Effectively, a “potential conflicts” set exists for every vehicle and those sets need to be kept current as time unfolds. This process is based on the data structure of the simulation system which is not this paper's issue. It is assumed that the set is given for every time step and we directly discuss the TTC computation part.

In the context of this paper, the intent of computing the TTC is to ascertain whether the currently planned trajectory is safe or not. If not, a safety-related adjustment will need to be made. For example, to avoid collisions with other vehicles including those on their own lane and others on the subject lane, the driver needs to judge whether the lane-changing acceleration will lead to conflicts with other vehicles or not. If a very short TTC value pertains with some vehicle, then the lateral movement is not acceptable. On the other hand, if the TTCs with all of the adjacent vehicles are satisfactory, the lane-changing maneuver can be perceived as safe and feasible.

However, this is a decision that is made after the TTC assessment has been performed. Hence, in this analysis, trajectory adjustment analysis is not considered although the procedures described here can be used to determine what adjustments would be appropriate.

In the TTC analyses that follow, the present or intended acceleration vector is assumed to be an input variable from the state of the system data or other functions. And that having been said, there are tests that pertain to the lateral accelerations being employed in the TTC analysis. For example, if the subject vehicle wants to change lanes then there should be acceleration in the lateral direction. If these tests reveal logical inconsistencies, then adjustments need to be made to the TTC analysis inputs before the assessment is conducted.

## 4. Methodology

The key to the method is a simple, straightforward way to determine when the “subject” vehicle will “touch” a “target” vehicle. As the reader might expect, the geometric shapes assumed for the two vehicles have a significant impact on the complexity of and time required for the calculations. Even though we assumed that the vehicles are basically rectangles when projected to the plane, we need to deal with the buffer areas in computing the TTC. Hence the shape used in the analysis should not be the physical rectangle. It should be larger or even a different shape.

Another reason to consider different shapes is that the geometric features of the shape will influence the complexity of the algorithm. In the following text, four algorithms are discussed based on different shapes.

### 4.1. Circle Algorithm

Circles are a valuable geometric shape with which to experiment. Determining when two circles touch is simply a matter of determining whether the distance between the circle centers is less than the sum of the radii. The problem is, the shape of the circles does not match well with our notion of a buffer. But that limitation does not imply that the use of circles should be omitted. This could be a valuable prescreening procedure.

**Figure 3:**
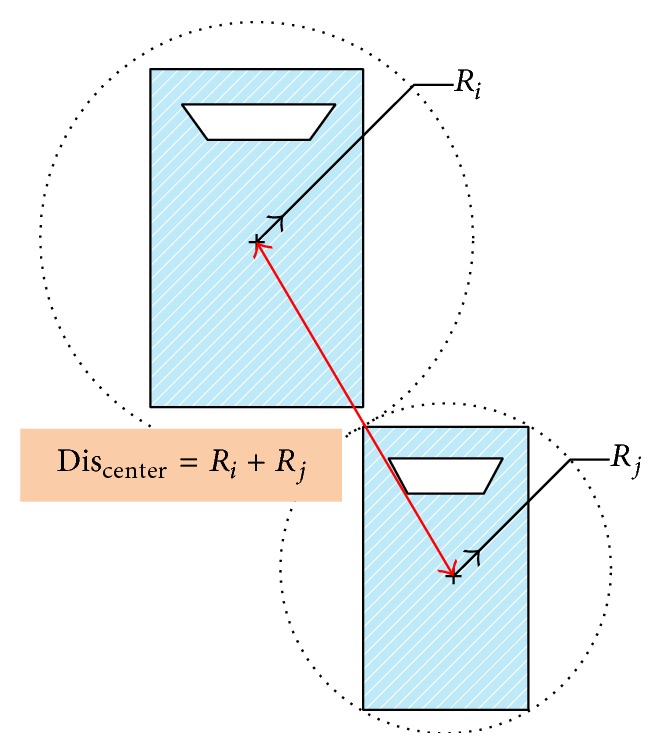
A collision based on circles as buffers.

As shown in Figure 3, if the buffers are circles, then, when the distance between the centers of two circles is equal to the sum of the two radii, the subject vehicle has touched the target vehicle.

Mathematically, given the radius, position, and state of motion for every vehicle (circle), the TTC is easy to obtain. The equation set is

(2) xi+vxi·TTCij+12axi·TTCij2 −xj+vxj·TTCij+12axj·TTCij22 +yi+vyi·TTCij+12ayi·TTCij2   −yj+vyj·TTCij+12ayj·TTCij22  =ri+rj2,

where TTC_*ij*_ is the unknown value of TTC between the *i*th and *j*th vehicle, *x*_*i*_ and *y*_*i*_ are the coordinates, *v*_*xi*_ and *v*_*yi*_ are speed components on *X* and *Y* direction, *a*_*xi*_ and *a*_*yi*_ are acceleration components on *X* and *Y* direction of *i*th vehicle, and *r*_*i*_  is the radius of the circle standing for the *i*th vehicle. It is a quartic equation with one unknown TTC_*ij*_. If there are real positive roots for the equation in the range of effective TTC values, the smallest value is the TTC. As can be seen, the computation is straightforward and simple.

### 4.2. Rectangle Algorithm

This algorithm treats both the subject vehicle and the target vehicles as rectangles. The rectangle for the subject vehicle is larger than its own physical rectangle to account for the buffer area. The target vehicle is represented by the rectangle of its own measure. When the two rectangles overlap, a collision has occurred.

In this case, it is not possible to solve a set of equations directly to compute the TTC. Simulation must be used. Hence, the algorithmic task is as follows: “given the orientation of two rectangles with certain speeds and accelerations, move them from one time step to another, and get the time when they overlap.”

**Figure 4:**
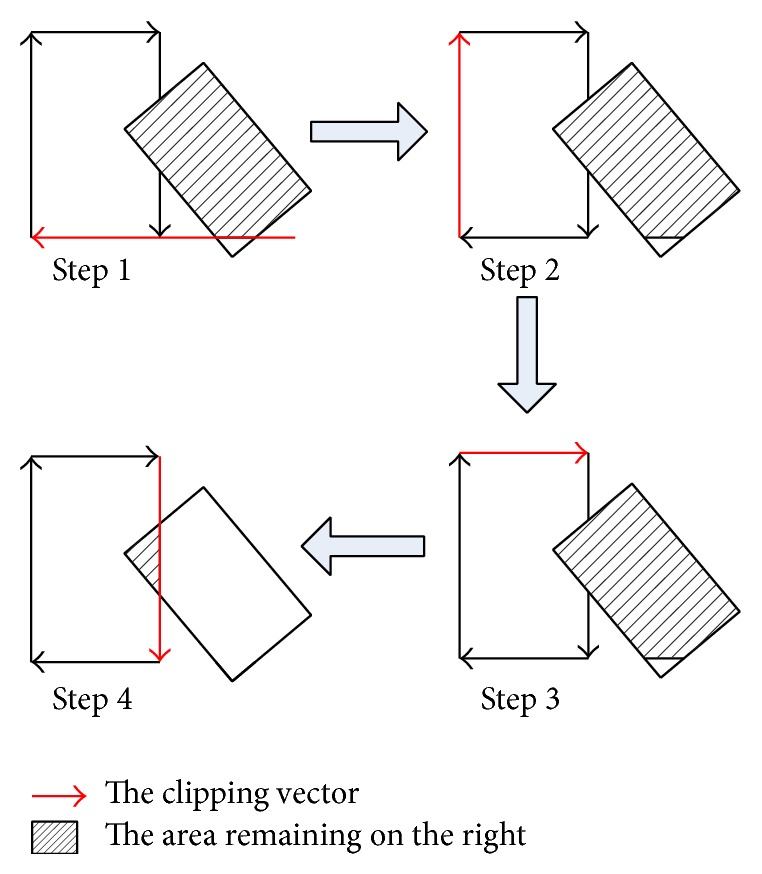
The process of the rectangle clipping algorithm.

Fortunately, it is trivial to locate a pair of rectangles in every time step of the simulation. A general polygon-clipping algorithm can be used to perform the detection. As shown in Figure 4, one of the two rectangles is defined to be the clipper and the other is the target. The edges of the clipper are assumed to be vectors oriented in a clockwise direction.

Each directed edge of the clipper is used to cut the target rectangle, retaining at each step the remaining piece of the target that is to the right of the clipping edge. This process continues until all the edges of the clipper have been examined. A collision exists if the target rectangle has a nonzero area remaining.

As each edge of the clipper is examined, the size of the remaining area is tested. To determine if an endpoint is to the right of the clipping vector, the cross product of that vector and the vector from the starting point of the clipping vector to the endpoint is applied. If one of two adjacent points is on the left while the other is on the right, the intersection point of the clipping vector and the line segment defined by those two points remains as a new endpoint of the rest piece.

### 4.3. Ellipse-Rectangle Algorithm

**Figure 5:**
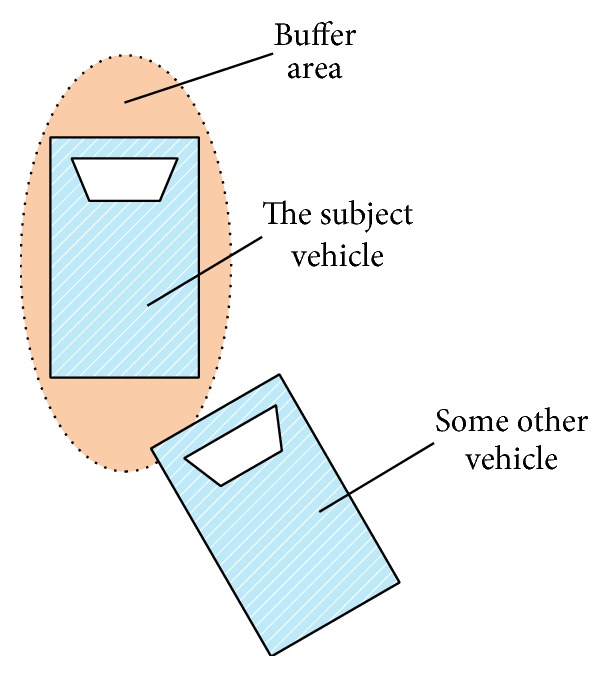
The illustration for the ellipse-rectangle algorithm.

As shown in Figure 5, this option uses an ellipse to represent the subject vehicle and a rectangle to represent the target vehicle. The ellipse around the subject vehicle is longer in the longitudinal direction than it is in the lateral direction. This ensures that the safety distance is longer in front and back than it is to the right or left.

A preliminary step is to set the dimensions of the ellipse. The values depend on the safety spacing retained by drivers. Although there are papers talking about various types of safety distance or safety headways, for driving behavior this kind of shielding area has been rarely researched. It is different from the minimum headway which provides enough stopping distance and clear vision. It should be smaller and, like the reaction time, varies among drivers and is difficult to determine by general trajectory data. Moreover it might be affected by the traffic condition and the speed. The higher the speed is, the larger the safety spacing should be.

However, the details of the dimensions of this ellipsoidal buffer area are not the main focus of this paper. It is sufficient to assume that the characteristics of the ellipse can be related to the dimensions of the vehicle. Thus, without loss of generality, we can set the major axis at 1.6 times the length of the vehicle and the minor axis at 1.3 times the width. Then the ellipse at every time step is defined by the following equation:

(3) 1ai2xitj−xcitj·cos⁡αitj+yitj−ycitj·sinαitj2 +1bi2yitj−ycitj·cos⁡αitj−xitj−xcitj·sinαitj2=1,

where *a*_*i*_ and *b*_*i*_ are the major and minor semiaxis of the *i*th vehicle's ellipse, *x*_*cit*_*j*__ and *y*_*cit*_*j*__ are the coordinates of centroid at *t*_*j*_, *α*_*it*_*j*__ is the angle of the vehicle's longitudinal axis with the positive direction of *x*-axis, and (*x*_*it*_*j*__, *y*_*it*_*j*__) is the coordination of any point on the ellipse.

As an aside, when doing the computations, a coordination transformation saves time. It pays to put the centroid of the ellipse at the origin of the coordinate system and align the axes with the orientation of the ellipse.

**Figure 6:**
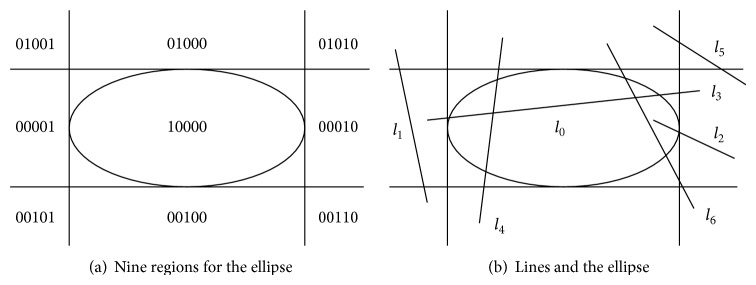
Detection of the intersection between the ellipse-rectangle and a line segment.

The next question to answer is this, “when do the ellipse and the rectangle overlap.” To identify the answer, the relationship of line segments to the ellipse needs to be examined. This is illustrated in Figure 6.

The process of identifying when the line segments intersect the ellipse involves the following steps, which are executed for each of the four line segments that comprise the rectangle for the neighboring vehicle.

**Step 1 .**

Divide the plane around the ellipse into 9 regions defined by the bounding box for the ellipse as illustrated in Figure 6(a). Assign an exclusive code to each region. Assign codes to the segment endpoints based on their relative locations.

**Step 2 .**

By “bitwise-or” and “bitwise-and” computations for the two endpoints of each line segment, identify which of the five conditions pertains to the spatial relationship between the line and the ellipse: (i) both segment endpoints are inside the ellipse as *l*_0_ in Figure 6(b); (ii) both points are on the same side of one edge of the bounding box like *l*_1_; (iii) one point is inside the ellipse while the other is outside as with *l*_2_; (iv) the two endpoints are on opposite sides of the ellipse as with *l*_4_; that is, one end is on the top while the other is on the bottom or one is on the left while the other is on the right. In this situation, there must be two intersection points: (v) conditions other than cases (i) to (iv), as with *l*_5_ or *l*_6_. For conditions (i), (iii), and (iv), the segment and ellipse overlap. For condition (ii), there is no overlap. If condition (v) pertains, the algorithm moves to Step 3.

**Step 3 .**

Since the ellipse and the line segment are both defined by equations, solve these equations simultaneously and see if a real-valued solution is identified that lies in between the endpoints. If so, the line segment and the ellipse overlap.

### 4.4. Comparing and Combining the Procedures

The above three algorithms all have advantages and disadvantages. Based on the performance of the three respects as approximation of the buffer area, accuracy, and the demand of numerical simulation, a comparison can be drawn between them. The result is shown in Table 1.

The circle algorithm is quick. No simulation is needed. But the circles do not reflect the shape of the buffer in any significant way unless circles of two different sizes are used: one longitudinally and one laterally. There can be situations when the circles intersect on the lateral side if the vehicles are in adjacent lanes. If a much smaller radius is used laterally, this drawback can be partly addressed. However, this can result in situations where the radius is too small and the TTC is too large.

The ellipse-rectangle procedure is good at representing the buffer area. Its accuracy is good, but simulation is needed to compute the TTC.

The rectangle algorithm is fair at representing the buffer area—areas outside the ellipse but within the buffer rectangle are really outside the buffer zone. But its accuracy is good. Simulation is necessary to obtain the TTC, but the clipping procedure makes it quick to determine at any given time step whether or not a collision has occurred.

Given the strengths and weaknesses of the three methods, we experimented with combining them. For example, if we start by using the circle procedure, no overlap arises in the future time intervals of interest; then no possibility of a collision exists. Hence, analysis at a finer level of detail is unnecessary. Similarly, if we use a small circle based on the inscribed circle of the original rectangle, if the two circles overlap, there must be a collision. Moreover, the exact moment at which the collision occurred has to be in between the time at which the big circles overlap and the time when the small circles first overlap.

**Figure 7:**
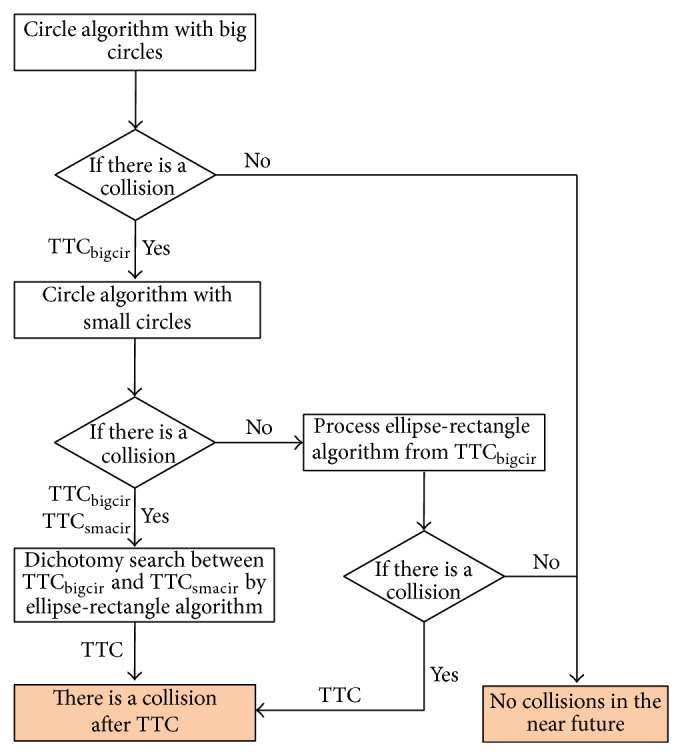
The combined algorithm.

As the ellipse-rectangle is the most rational and accurate among the three it should be used to determine the exact value of TTC. Based on these ideas, the algorithm shown in Figure 7 was developed.

The combined algorithm starts by using the big circle algorithm. The idea is to use the circle algorithms to narrow the time range of the simulation so that computation time can be saved.

Assume that the current time is *t*_0_. If no overlap between the subject and target circles is identified beyond *t*_0_, then no collision is going to arise (highlighted at the bottom of Figure 7). If the big circle algorithm identifies a time of overlap, it is designated as *t*_0_ + TTC_bigcir_.

If *t*_0_ + TTC_bigcir_ is nonzero, then a small circle test ensues. Moreover, if an overlap is identified in this case, that time is denoted as *t*_0_ + TTC_smacir_. If *t*_0_ + TTC_smacir_ is nonzero, then there must be a collision between *t*_0_ + TTC_bigcir_ and *t*_0_ + TTC_smacir_. To find out the exact TTC, the ellipse-rectangle algorithm is employed.

If a collision is predicted based on the big circle analysis, while none is predicted by the small circle one, simulation is used to see if a collision occurs, but the simulation starts at *t*_0_ + TTC_bigcir_. Values between *t*_0_ and *t*_0_ + TTC_bigcir_ can be ignored.

One important issue is the determination of the geometric dimensions. The size of the ellipse is the same as was discussed previously.

The radius of the big circle should be greater than or equal to the sum of the major semiaxis of subject ellipse and the semidiagonal length of the rectangle. This is because the function of the big circle algorithm is to find those vehicle pairs which have no chance of colliding. The radius of the small circle should be the same length of the smallest axis of the ellipse or rectangle.

## 5. Numerical Example and Comparison

A computational procedure was developed in MATLAB which implemented the analysis procedure shown in Figure 7. The efficiency of the procedure was tested and the results are presented in this section. The procedure can be applied in an environment which treats lateral movements as continuous in space. The buffer areas are represented by ellipses. If there is a need to use the rectangle to substitute the subject vehicle then in the algorithm process, for example, to obtain the exact TTC as a conflict intensity indicator, the ellipse-rectangle algorithm is replaced by the rectangle algorithm.

### 5.1. Data Process

The data we used to validate the simulation system as well as this algorithm is the dataset of vehicle trajectory data completed as part of the Federal Highway Administration's (FHWA) Next Generation Simulation (NGSIM). The data analyzed in this paper represent vehicle trajectories on a segment of U.S. Highway 101 (Hollywood Freeway) in Los Angeles, California, collected between 7:50 a.m. and 8:05 a.m. on June 15, 2005. The NGSIM trajectory dataset provides longitudinal and lateral positional information for all vehicles in certain spatiotemporal regions. We applied a symmetric exponential moving average filter (sEMA) to smooth the data for all trajectories before any further analysis. After that, velocity and acceleration values were decomposed into vectors combining *x*-axis and *y*-axis components.

### 5.2. Test and Results Comparison

The tests were performed on a standard desktop computer using MATLAB (R2012a). We set the upper bound on the TTC as 5 seconds. A total of 327616 vehicle pairs were tested. Several comparison experiments were conducted using the same dataset. Some of the results were selected at random to test the correctness of the result. For example, the vehicle trajectories were plotted to ensure that the TTCs correctly reflected what would happen to the vehicles given their trajectories.

Comparisons were drawn among the aforementioned algorithms, a process using big circle and ellipse-rectangle without small circle (numerical simulation starting from TTC_bigcir_) and a process using small circle and ellipse-rectangle only (dichotomy search between *t*_0_ + TTC_bigcir_ and *t*_0_ + TTC_smacir_). Two indicators were compared among all those algorithms. First is the computation time and the second is the correlation coefficient between inverse TTC and reaction intensity which is measured by the deceleration vector and steering angle change. The optimized algorithm using both small and big circles is the most efficient algorithm. And the last 4 algorithms in Table 2 have the same TTC result (since the TTC values are all determined by the ellipse-rectangle model). The correlation coefficient of those is larger than the first 2 as the ellipse-rectangle model is more reliable than the circle model and the rectangle model. It could be asserted that the algorithm using both small and big circles is the best one.

As can be seen, the optimized algorithm provides the shortest computation time. Such an algorithm could also be applied to traffic accident monitoring or safety analysis in which the ellipse-rectangle representing geometry might be replaced by the rectangle with the same dimensions of the vehicle. There are still several problems to be solved as the exact parameters of the ellipse and the specific relationship between TTC and response acceleration stimulated by it. Future work should be done to explore the importance of and to quantify the consequence of TTC.

## 6. Conclusions

The optimized algorithm is proposed to calculate TTC in simulation model of freeway traffic. The efficiency has been proved sufficient for the simulation. It could also be applied in other traffic conditions when the shape of vehicle could not be neglected. In the future, the TTC got from this algorithm should be tested and validated in the context of the entire traffic simulation.

## Figures and Tables

**Table 1:** Comparison of the three algorithms.

Type of algorithm	Buffer area representation	Accuracy	Simulation necessary
Circle	Not good	Not good	No
Rectangle	Fair	Good	Yes
Ellipse-rectangle	Good	Good	Yes

**Table 2:** Computation times for the example dataset.

Algorithm type	Computation time (sec)	Correlation coefficient between inverse TTC and reaction intensity *r* (*P* < 0.01)
Circle algorithm	18	0.381
Rectangle algorithm	32	0.522
Ellipse-rectangle algorithm	45	0.732
Optimized algorithm	17	0.732
Optimized algorithm only using big circle	36	0.732
Optimized algorithm only using small circle	48	0.732
